# The Association Between Maternal Asthma and Child Autism: A Systematic Review and Meta‐Analysis

**DOI:** 10.1002/aur.70071

**Published:** 2025-06-20

**Authors:** Renee van der Schaaf, Vanessa E. Murphy, Soriah Harvey, Paige Dent, Alison Lane, Olivia Whalen

**Affiliations:** ^1^ School of Psychological Sciences University of Newcastle Newcastle New South Wales Australia; ^2^ Healthy Minds Research Program Hunter Medical Research Institute Newcastle New South Wales Australia; ^3^ School of Medicine and Public Health University of Newcastle Newcastle New South Wales Australia; ^4^ Asthma and Breathing Research Program Hunter Medical Research Institute Newcastle New South Wales Australia; ^5^ Olga Tennison Autism Research Centre La Trobe University Melbourne Victoria Australia

**Keywords:** asthma, asthma medication, autism, child, infant, maternal, pregnancy

## Abstract

Maternal asthma has been linked to child autism. In this study, we systematically reviewed observational studies published between July 2001 and February 2024 that assessed maternal asthma during pregnancy (exposure) and child autism (outcome). Databases searched included MEDLINE, CINAHL, EMBASE, and PsycINFO. Of the 350 potential studies, 19 met the inclusion criteria (2,530,716 participants; 73,065 autistic participants). Quality was assessed with the Newcastle–Ottawa Scale. Meta‐analyses using proportions and odds ratios were conducted using the Mantel–Haenszel method with a random‐effects model. Compared to women without asthma, there was an increased odds of child autism with any history of maternal asthma (OR = 1.32; 95% CI = 1.21, 1.44; *I* ^2^ = 61%, *n* = 14), with current asthma during pregnancy (OR = 1.23; 95% CI = 1.12, 1.35; *I* ^2^ = 35%, *n* = 10) and with medication use during pregnancy (OR = 1.48; 95% CI = 1.30, 1.68; *I* ^2^ = 0%, *n* = 3). However, when women with asthma who used asthma medication were compared to those with asthma who did not use medication, there were no increased odds for child autism (OR = 1.07; 95% CI = 0.89, 1.27; *I* ^2^ = 34%, *n* = 2). Maternal asthma is associated with an increased odds of child autism. Future studies should consider neurodivergence in the parents, the severity of asthma, and the effectiveness of prescribed medication in managing the mother's asthma to improve our understanding of this association.

**Trial Registration:** PROSPERO registration: CRD42021265060

**Summary**

- This study systematically reviewed and meta‐analyzed studies that looked at the relationship between a mother's asthma and her child's autism.
- We found that women with asthma may be more likely to have an autistic child, regardless of whether they used asthma medication during pregnancy.
- However, the studies did not consider parental neurodivergence, which could influence the findings considering the high heritability rate of autism.
- Additionally, the severity of the mother's asthma and the effectiveness of the prescribed medication in controlling her asthma is not known.
- Therefore, these results are interpreted with some caution and will influence future research methodology that examines the relationship between maternal asthma and child autism.

## 1 Introduction

Maternal immune activation, occurring when acute or chronic inflammation activates the mother's immune system during pregnancy, has been implicated as a mechanism involved in infant neurodevelopmental differences, including autism (Han et al. 2021). Asthma is the leading immune disease to complicate pregnancy, occurring in 8%–12% of pregnancies worldwide (Hansen et al. 2013; Sawicki et al. 2011). Changes in asthma are unpredictable during pregnancy, which may result in maternal and placental physiological changes resulting in adverse perinatal outcomes (Murphy et al. 2005). Results from meta‐analyses show that infants born to mothers with asthma are at 36% increased odds of congenital malformations (Robijn et al. 2024). In addition, there is a 25% increased risk of respiratory distress, a 27% increased risk of neonatal hospitalization at birth, and a 14% increased risk of perinatal mortality for infants born to mothers with asthma, compared to infants born to mothers without asthma (Robijn et al. 2024). Although Robijn et al. (2024) did not evaluate the impact of asthma severity, exacerbations, or asthma medication use on perinatal outcomes, oral corticosteroid use is more commonly associated with adverse perinatal outcomes (Davies et al. 2020) such as preterm birth (Schatz et al. 2004), low birth weight (Rocklin 2011; Schatz et al. 2004) and congenital malformations (Rocklin 2011). Furthermore, discontinuation of asthma medication during pregnancy is associated with preterm birth (Davies et al. 2020). Considering the influence maternal asthma has on perinatal health outcomes, it is important to also consider whether maternal asthma influences neurodevelopmental outcomes.

Asthma is the most reported maternal immune condition among autistic children, particularly among autistic boys (Patel et al. 2020). Moreover, immune‐mediated conditions such as asthma, allergy, eczema, and atopic dermatitis occur at a higher rate in autistic individuals, implying that allergic inflammation may have an important role in autism (e.g., Magalhães et al. 2009). Yet, allergic and neurodevelopmental conditions share common mechanisms that suggest a bi‐directional relationship may be at play. Inflammation, immune dysregulation, gene expression, epigenetics, mitochondrial dysfunction and environmental influences are mechanisms implicated in each of these co‐occurring conditions (Chua et al. 2020). Additionally, adverse perinatal outcomes associated with maternal asthma (e.g., preeclampsia, preterm birth, and low birth weight) have been independently linked to neurodevelopmental differences in the child, such as autism (e.g., Mann et al. 2010). Gordon et al. (1970) published evidence suggesting infants born to mothers with asthma have a higher rate of neurological differences at 1 year of age compared with children born to mothers without asthma, indicating that the fetus in utero exposure to maternal asthma may play an important role in their neurodevelopmental trajectory. However, it was not until the early 2000s that this association was further investigated, and autism was more likely to be observed in the infants of women who experienced asthma during the second trimester of their pregnancy (Croen et al. 2005). Autism is one form of neurodiversity that affects 1 out of every 100 children worldwide (Zeidan et al. 2022). Autistic characteristics or traits occur across a broad spectrum and are often discussed in reference to the expected neurodevelopmental trajectory of children. To avoid stigmatizing language that may come with such comparisons, we refer to this as neurodevelopmental differences.

In 2018, our team published the first systematic review examining the relationship between maternal asthma and child cognitive and behavioral outcomes (Whalen et al. 2018). Of the 10 studies included, 6 reported on autism diagnosis (Croen et al. 2005; Langridge et al. 2013; Leonard et al. 2006; Lyall et al. 2014; Micali et al. 2004; Mouridsen et al. 2007). However, only one found the association between maternal asthma and child autism to be statistically significant after adjusting for maternal and sociodemographic characteristics (Croen et al. 2005). Another of the studies that investigated autism characteristics found that asthma or allergy in mothers was associated with some social characteristics of autism that require a higher level of support (Patel et al. 2017). The other studies found no association between maternal asthma and child autism (Langridge et al. 2013; Lyall et al. 2014; Micali et al. 2004; Mouridsen et al. 2007), neurodevelopmental disorders (Flannery and Liederman 1994), developmental delay (Lyall et al. 2014), or autism with an intellectual disability (Leonard et al. 2006). Overall, the systematic review identified that there was a lack of prospective studies, and discrepancies across the study designs meant a meta‐analysis was not feasible.

More recently, two systematic reviews have examined the relationship between maternal conditions in pregnancy (e.g., asthma, allergy, diabetes, or eczema/atopic dermatitis) and child neurodevelopmental conditions such as autism, attention deficit hyperactivity disorder (ADHD) and Tourette syndrome (Han et al. 2021; Seker et al. 2023). As neither review completed a meta‐analysis, the authors were unable to quantify the effects of maternal asthma on child autism. However, after considering the effect estimates of individual studies, they concluded that maternal states associated with inflammation were related to an increased likelihood of neurodevelopmental differences in children (Han et al. 2021), supporting the hypothesis that maternal immune activation could be linked with child neurodevelopmental differences (Seker et al. 2023). Furthermore, this association was more apparent when asthma was present during pregnancy, particularly in the first and second trimesters (Seker et al. 2023). Therefore, emerging research indicates there may be an association between maternal asthma and child autism. With asthma occurring commonly in pregnancy, and the prevalence of autism increasing in the population, there is a need for more research into associations between maternal asthma and child autism. The aim of this study was to synthesize the recent and available evidence and quantify via meta‐analyses any association between maternal asthma during pregnancy and child autism (i.e., when the child is autistic).

## 2 Methods

### 2.1 Study Selection and Search Strategy

The protocol for this systematic review and meta‐analysis was registered at PROSPERO (CRD42021265060) and was conducted according to the Preferred Reporting Items for Systematic Reviews and Meta‐Analyses (PRISMA) statement (Page et al. 2021). Electronic searches of MEDLINE, CINAHL, EMBASE, and PsycINFO were conducted using the search string: “Pregnan* OR gestation* OR obstet* OR maternal” *AND* “Asthma* OR Wheez*” AND “autism spectrum disorder OR autism OR Asperger syndrome OR autistic disorder OR pervasive developmental disorder.” Results were limited to human studies that were published in English between July 1, 2001 and February 1, 2024. The search was first conducted in July 2021 to include all studies within the last 20 years, with an update to the search being conducted in February 2024. Manual searches of the reference lists of included studies and excluded systematic reviews were conducted (snowballing). The papers were uploaded into Covidence (Innovation 2023) for title and abstract screening, and full‐text screening.

### 2.2 Eligibility Criteria

One primary reviewer (S.H. or R.v.d.S.) and a secondary reviewer (V.E.M. or O.W. or P.D.) independently assessed the papers against the inclusion criteria. For inclusion, publications: (1) were an original study in humans (cohort, cross‐sectional, case–control, randomized controlled trials, and clinical trials); (2) had the full text available in English; (3) included whether the child is autistic, as well as a comparison non‐autistic group; and (4) included the incidence of maternal asthma for the autism group and the comparison group. The outcome of interest, autism, was defined by either a clinical diagnosis, parent‐reported diagnosis in combination with researcher observed autism in the diagnostic range, medical database classification under the Diagnostic and Statistical Manual of Mental Disorders 5th Edition (American Psychiatric Association 2013), or criteria including previous categories and diagnostic labels used to identify autism (inclusive of Autism Spectrum Disorder, Autistic disorder, Asperger's syndrome and Pervasive developmental disorder not otherwise classified) according to the Diagnostic and Statistical Manual of Mental Disorders 4th Edition (American Psychiatric Association 2000). Autism recorded according to the International Classification of Diseases (ICD) 9th (World Health Organization 2011) and 10th (World Health Organization 2016; ICD‐10) revisions was also included. The exposure of interest was defined as either physician‐diagnosed asthma in the mother prior to or during pregnancy, asthma symptoms and/or medication use in the past 12 months (occurring prior to the birth of the child), or asthma determined from ICD‐10 codes in database studies. Studies were excluded if they were gray literature (e.g., conference abstracts and theses), letters to the editor, review papers, case studies, qualitative studies, statistical modeling papers, in vitro/animal studies, or papers that did not include data on maternal asthma incidence or autism in the child. Conflicts were discussed between two reviewers (R.v.d.S., S.H., O.W., V.E.M., or P.D.) until consensus was reached.

### 2.3 Quality Assessment

Study quality was assessed independently by two reviewers (S.H., R.v.d.S., O.W., or P.D.). The Newcastle–Ottawa scale (NOS) tool was utilized for cohort or case–control studies (Wells et al. 2014). For comparability, a study was awarded a star for controlling for the child's age and sex, and an additional star for controlling for other confounders, such as maternal age, ethnicity, plurality, education level, geographic location, or maternal smoking. None of the studies controlled for autistic characteristics in the parents, or for autism among the family members. The study was then categorized as being good, fair, or poor quality based on the Agency for Healthcare Research and Quality (AHRQ) system of points awarded for stars via threshold conversion and quality (Supporting Information S1).

### 2.4 Data Extraction

Data were extracted from each study by one reviewer (R.v.d.S. or S.H.) and then independently checked by a second reviewer (V.E.M., O.W., or P.D.). The data extracted included first author, date of publication, study location, sample size, study design, child age, study aim/s, adjustment for covariates, measurement of exposure, measurement of outcome, asthma management details, exposure to maternal cigarette smoking, maternal comorbidities, family ethnicity, maternal age at baseline, and statistical output and proportions in the autism and non‐autism groups. Conflicts were discussed between the two reviewers until consensus was reached. The studies were only included if the required data was available in the published paper or supplemental material. The corresponding authors were not contacted for additional information.

### 2.5 Analysis

Data extracted contributed to four meta‐analyses: (1) examined the relationship between any history of maternal asthma and child autism; (2) examined the association between current maternal asthma during the pregnancy and child autism; (3) and (4) examined asthma medication use during pregnancy and child autism comparing women with asthma using medication with women with asthma not using medication (3); and with women without asthma (4). Review Manager 5.0 (Review Manager (RevMan) 2020) was used to conduct quantitative meta‐analyses using the Mantel–Haenszel method with a random‐effects analysis model. Stata (StataCorp 2023) was used to conduct quantitative meta‐analyses with odds ratios (ORs), and to assess publication bias using Egger's test. Substantial publication bias was confirmed when the funnel plot was considered asymmetric, and Egger's test was significant at the 0.05 level (Egger et al. 1997). Funnel plots and Egger's test statistics are not appropriate for meta‐analyses with less than 10 studies due to low power (Page et al. 2023). Heterogeneity of the pooled estimate was assessed using the *I* ^2^ index, categorized into three levels: mild (< 25%), moderate (25%–50%), or high (> 50%) variability between the studies (Higgins et al. 2003). A sensitivity analysis was performed by omitting one study at a time and recalculating the summary OR to determine the influence of each study on the summary estimate. Further sensitivity analyses with all studies assessed as poor quality removed was also conducted. All sensitivity analyses are in Supporting Information S1. ORs were calculated with 95% confidence intervals (CIs) and an alpha of 0.05.

## 3 Results

### 3.1 Literature Search

**FIGURE 1:**
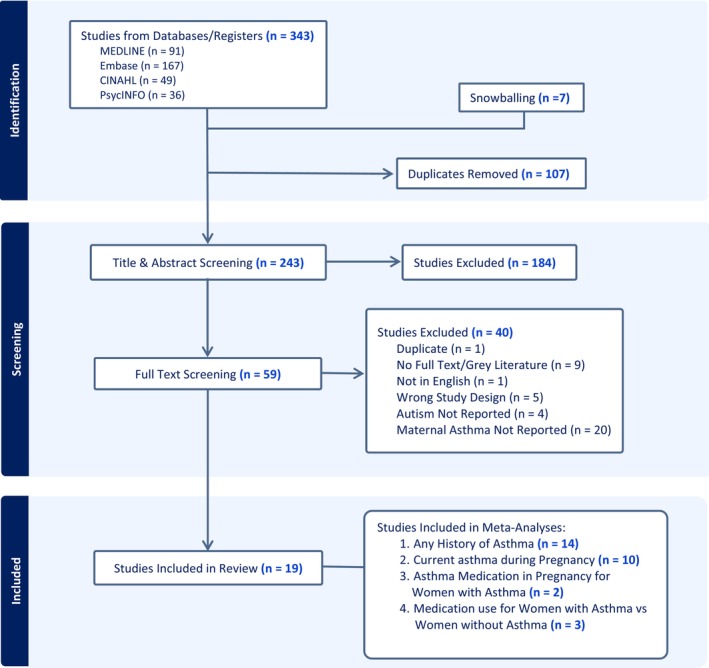
PRISMA flow diagram showing the selection of studies examining the association between maternal asthma and the odds of child autism.

A search of the online databases identified 343 potential studies, with a further 7 studies being identified through snowballing. After removing duplicates (*n* = 107), 243 studies underwent title and abstract screening (*n* = 184 excluded). Full‐text screening was conducted on the remaining 59 studies (*n* = 40 excluded). The remaining 19 studies were deemed suitable for inclusion in the systematic review, as shown in Figure 1.

### 3.2 Characteristics of Included Studies

These 19 studies included a total of 2,530,716 participants (73,065 autistic participants). Table 1 provides an overview of the study characteristics. Seven of the studies were cohort studies, and 12 were case–control studies. All studies were conducted between 2004 and 2023 in Australia (Langridge et al. 2013; Leonard et al. 2006), the United States (Carter et al. 2023; Croen et al. 2023, 2011, 2005, 2019; Grivas et al. 2022; Hisle‐Gorman et al. 2018; Lyall et al. 2014; Singer et al. 2016; Yu et al. 2023), Denmark (Gidaya et al. 2016; Mouridsen et al. 2007; Singer et al. 2017; Su et al. 2017), Ireland & New Zealand (Casey et al. 2022), Sweden (Gong et al. 2019), and the United Kingdom (Micali et al. 2004).

**TABLE 1:** Characteristics of the 19 studies included in the systematic review.

First author (year)	Country	Study design	Period	Total sample size (*n*)	Child age	Ascertainment of asthma	Ascertainment of autism	Race	Confounders adjusted for	NOS AHRQ rating	Association between maternal asthma and child autism
Carter 2023^a^	USA	Cohort	Infants born 2001–2004	308,536 (5131 autism; 303,405 controls)	Birth: 5 years	Asthma during pregnancy was identified by ICD‐9 code 493 and at least one prescription for an asthma‐specific medication.	Electronic medical record entries using ICD‐9 codes or ICD‐10 for autistic disorders, Asperger's syndrome, and pervasive developmental disorders not otherwise specified.	White: total with autism = 21%; total controls 24%	Maternal age, parity, self‐reported race/ethnicity and education, census tract‐level household income at child's first birthday, maternal smoking during pregnancy, maternal mental health conditions during pregnancy (any anxiety, depression, or use of selective serotonin reuptake inhibitors), and history of comorbidity (≥ 1 diagnosis of heart, lung, kidney, liver disease, or cancer) and child's sex.	Good	Maternal asthma associated with higher odds of autism with or without gastrointestinal disturbances, yet was highest for those with gastrointestinal disturbances.
Casey 2021	Ireland and New Zealand	Cohort	Data collected November 2004 to February 2022	63 (25 autism; 38 Controls)	Birth: 6 years	Physician diagnosed asthma	Children in Cork were considered autistic if diagnosis was made by a professional (Early Intervention Service or child psychiatrist). In Auckland, autism identified via parent report.	White: 92% of both groups	NA	Poor	Differences in IL‐17A expression were observed at 20 weeks of gestation among mothers of autistic children.
Croen 2005	USA—Northern CA	Case–control	Infants born 1995–1999	2502 (407 autism; 2095 controls)	6–10 years	Diagnosed at inpatient and outpatient visits in the period of 2 years preceding delivery through 2 years following delivery (*ICD‐9‐CM* codes available from authors).	At least one diagnosis meeting the International Classification of Diseases, Ninth Revision, Clinical Modification (ICD‐9‐CM) criteria for autism, Asperger's disorder, or pervasive developmental disorder not otherwise specified, was identified by electronically scanning the KPMCP clinical databases	White: autism = 51.6%; controls = 45.1%	Maternal age, maternal race/ethnicity, maternal education, infant sex, plurality	Fair	After adjustment for maternal factors, maternal asthma remained significantly associated with child autism.
Croen 2011	USA	Case–control	Infants born 1995–1999	575 (291 autism; 284 controls)	Birth: 7 years	Maternal asthma identified from inpatient and outpatient databases and abstracted from prenatal records.	At least one diagnosis of autism using the ICD‐9 classifications, including Asperger's Disorder or Pervasive Developmental Disorder Not Otherwise Specified. Children with fragile X syndrome, tuberous sclerosis, or neurofibromatosis were excluded.	White: autism = 54%; controls = 49%	NA	Good	Prolonged maternal exposure to terbutaline (> 2 days) in the third trimester is associated with an increased odds of child autism. However, the small sample size resulted in an imprecise effect estimate. B2AR agonist use during pregnancy did not explain the authors' previous observation of an increased odds of child autism among mothers with asthma (Croen et al. 2005).
Croen 2019	USA—CA, CO, GA, Maryland, NC, PA	Case–control	Infants born 2003–2006	1578 (663 autism; 915 controls), and the 984 participants in Developmental Delay group were not included in this review	2–5 years	Asthma during pregnancy determined based on diagnosis prior to child's date of birth, or medication used for asthma during pregnancy. Any lifetime history of asthma determined up until data collection, including after baby's birth (asthma identified via computer‐assisted telephone interview with primary caregiver, medical forms, survey, or prenatal medical records).	Face‐to‐face standardized developmental assessment for autism using multiple tools (ADOS and ADI‐R).	White: autism = 61%; controls = 74.5%	Maternal age and education at date of delivery of child, maternal race‐ethnicity, household income at time of caregiver interview, and child sex.	Good	Maternal asthma occurred in 25%–30% of women and occurred significantly more often among mothers of autistic children compared with the general population. Those who received asthma treatment during pregnancy had an increased odds of child autism.
Croen 2023	USA	Case–control	Infants born 2011–2016	1278 (311 autism; 967 controls)	3–8 years	Clinician diagnoses were identified from the maternal inpatient and outpatient electronic health records; ≥ 2 dispensed medications during pregnancy, or asthma treatment during pregnancy with asthma history.	Autism identified using the DSM‐IV or DSM‐5 criteria, and had an autism diagnosis recorded on their electronic medical records on at least one occasion; 80% were diagnosed at a KPNC autism evaluation center by a multidisciplinary team using a standardized protocol, including the Autism Diagnostic Observation Schedule (27). The remaining 20% were diagnosed by developmental‐behavioral pediatricians, child psychiatrists, pediatric neurologists, or general pediatricians.	White: 45% of the sample	Child sex, birth year, maternal age, maternal race, and maternal education	Good	Maternal asthma is significantly associated with increased odds of child autism. Mothers with both asthma and obesity had the highest odds of child autism among female children.
Gidaya 2016^a^	Denmark	Case–control	Infants born 1997–2006	57,200 (5200 autism; 52,000 controls)	10–16 years	Inpatient and outpatient maternal asthma was identified using ICD‐8 and ICD‐10 codes listed by the authors, and they were present in the register at any time before the delivery of the child.	ICD‐10 classifications of childhood autism, atypical autism, Asperger syndrome, and pervasive developmental disorder‐unspecified.	NA	Parental age and gender of the child	Good	Autistic children were more likely than controls to be male, have a higher parental age, and have a mother with an asthma diagnosis before the birth of the child. However, the association between maternal asthma and child autism was not statistically significant. Exposure to B2AR agonists during the prenatal period was associated with increased odds of child autism compared with those who were unexposed.
Gong 2019	Sweden	Case–control (nested case–control design in a population‐based cohort)	Infants born 1992–2013	251,834 (22,894 autism; 228,940 controls)	15.4 years	Records from any of the three registers, that is, the Medical Birth Register (MBR), National Patient Register (NPR), and the Swedish Prescribed Drug Register (SPDR) to identify asthma. Tick box for asthma/lung diseases at the first antenatal visit OR primary diagnosis of asthma for outpatient visits or hospitalizations OR two or more dispenses of ICS, LTRA, SABA. Therefore, this includes intermittent asthma, including childhood asthma or asthma in later adulthood (after birth of the baby).	Diagnosis of autism was recorded according to the International Classification of Diseases 9th and 10th revisions.	Country of birth is Sweden = autism 83.8%; controls 84%	Parity, maternal smoking during pregnancy and civil status at year of child birth, country of birth and age at child birth for mothers and fathers, highest education between parents, and maternal body mass index at first antenatal visit.	Good	Increased odds of child autism among mothers with asthma. The association between maternal asthma remained similar in magnitude within extended families, suggesting that genetic and environmental factors shared by half‐siblings and cousins did not seem to confound the association with maternal asthma. No statistically significant difference in the odds of child autism for women with asthma who were medicated with β2‐agonists or other asthma medications during pregnancy compared to those without medications.
Grivas 2022	USA	Cohort	Infants born 2000–2010	123,817 (1258 autism; 122,559 controls)	Birth: 5 years	Asthma identified using the International Classification of Diseases, version 9 (ICD‐9), National Drug Code (NDC), and current procedural terminology (CPT) codes.	A child was considered to have a diagnosis of autism if they had at least two separate ICD‐9 diagnostic claims of autistic disorder, Asperger syndrome, or unspecified pervasive developmental disorder, and no diagnostic claims for childhood disintegrative disorder or Rett syndrome.	White: High prevalence COC clusters = 69%; dd/seizure COC clusters = 71%; low prevalence COC clusters = 77%; controls = 73%	NA	Poor	Maternal asthma was significantly associated with increased odds of autism with co‐occurring developmental delay and seizures.
Hisle‐Gorman 2018	USA Military Health System Database	Case–cohort study	Identified between 2000 and 2013	35,040 (8760 autism; 26,280 controls)	2–18 years	Prenatal diagnoses in mothers' inpatient and outpatient records. Mothers' ambulatory pharmaceutical records identified medications prescribed during the 3 months preceding pregnancy and the pregnancy period. ICD‐9 codes.	Identified on the Military Health database, on at least two separate encounters, as being autistic according to the International Classification of Diseases, Ninth Revision.	NA	Active‐duty status (military service), total maternal healthcare visits in the year before the child's birth	Good	Mothers with an asthma diagnosis and those with an asthma diagnosis who are prescribed pharmaceutical treatment have significantly increased odds of child autism.
Langridge 2013^a^	Aus (WA)	Cohort	Infants born 1984–1999	377,718 (1179 autism; 376,539 controls); and the group of 4339 cases of intellectual disability without autism were not included in this review	6–22 years	Not described. Could be the Midwives notification system which has demographic information about the mother including medical conditions.	Diagnosis of autism in three main electronic disability databases used in Australia. All diagnoses verified by a Central Diagnostic Panel (from 1991) and cross‐disciplinary reporting protocols from 1997.	NA	Models used adjusting for a multitude of different variables	Good	After adjusting for important confounders, maternal asthma was not associated with increased odds of child autism or autism with intellectual disability.
Leonard 2006^a^	Aus (WA)	Cohort	Infants born 1983–1992	237,155 (191 autism with intellectual disability; 236,964 control without intellectual disability)	Birth: 16 years	Online data linkage—All medical conditions were coded using the International Classification of Diseases, Ninth Revision (ICD‐9), and pre‐existing medical conditions recorded on the midwives' notification form.	NA	NA	Infant sex and birth order, maternal ethnicity, age group, marital status, height, country of birth, health insurance status, and paternal occupation, as well as the Accessibility/Remoteness Index of Australia, as an indicator of geographic remoteness from major service centers.	Good	Maternal asthma is not associated with child autism and intellectual disability.
Lyall 2014	USA	Case–control	From 2006	951 (560 autism; 391 controls)	2–5 years	Environmental Exposures Questionnaire (EEQ), an Autoimmune Survey (AIS), a family medical history (FMH), and when available, medical records (prenatal).	The Autism Diagnostic Observation Schedule (ADOS) and Autism Diagnostic Interview Revised (ADI‐R) were administered by research team after children identified via the California Department of Developmental Services (DDS) records.	Autism group = 59% Caucasian	Maternal age	Good	Mothers with asthma did not have increased odds of child autism.
Micali 2004	UK	Case–control	Data collected July 1998 to June 1999	301 (95 autism; 206 controls)	5–6 years	Self‐report and validation of self‐reports via medical records, after parents were asked permission to contact their General Practitioners (GP). Eight declined to consent for access to GP records.	Intensive screening including an initial screening, 2‐week comprehensive developmental assessment, and then administration of the ADI‐R for those suspected of being autistic.	NA	NA	Good	There was no association between maternal asthma and child autism. However, small sample sizes in each group reduced power to detect an effect.
Mouridsen 2007	Denmark	Case–control	Infants born 1960–1984	441 (111 autism; 330 controls)	5.4 years	Medical records—diagnoses are listed as ICD‐10 diagnoses	Medical records in accordance with ICD‐9.	NA	NA	Good	Maternal asthma was not associated with a higher proportion of child autism.
Singer 2016	USA	Case–control	Infants born 2003–2006	1173 (463 autism; 710 controls)	30–68 months	The maternal medical history questionnaire and the family autoimmune disease survey. A mother was categorized as having maternal asthma during pregnancy if age of onset was recorded and was prior to the child's delivery date or unknown, or if she reported medication or treatment for asthma during pregnancy.	The Social Communications Questionnaire (SCQ) was used. Children with an SCQ score below 11 and without a previous autism diagnosis were asked to participate in a general developmental evaluation in the clinic using the Mullen Scale of Early Learning (MSEL). If the SCQ score was above 11, the child had previously received an autism diagnosis, or a clinician suspected autism during the clinic visit. The child additionally received a full autism evaluation that included the Autism Diagnostic Observation Schedule (ADOS).	White: autism = 62%; controls =76%	Maternal race, maternal education, current household income at time of questionnaire, maternal age at birth, parity, active smoking during pregnancy, maternal psychiatric condition history, and child's sex.	Poor	There were no increased odds of autism with environmental asthmagen exposure; this result was also seen when data was stratified for maternal asthma.
Singer 2017^a^	Denmark	Case–control	Infants born 1993–2007	59,915 (11,869 autism; 48,046 controls)	10–24 years	Maternal asthma defined as a history of either a maternal asthma diagnosis or a dispensed asthma medication. Identified via the National Patient Register for medical diagnoses, using ICD‐8 or ICD‐10 diagnosis in the Danish National Patient Register, which contains medical diagnoses by specialists, and this was prior to the date of delivery.	Reported diagnosis of autism according to the ICD‐10 codes identified by linking the Danish Psychiatric Central Register (DPCR) for information on psychiatric diagnoses and the National Patient Register for medical diagnoses.	NA	Child's year of birth, child's sex, maternal age at birth, paternal age at birth, parity, total parental income during the occupational year, highest parental education as of the occupational year, psychiatric diagnosis for either parent prior to the child's birth, maternal smoking at some point in the pregnancy, urbanicity of birthplace, parent was born in Denmark (yes, no).	Good	Maternal occupational asthmagen exposure had a weak inverse association with child autism, while there was no association between paternal occupational exposure and autism.
Su 2017^a^	Denmark	Cohort	Infants born 1997–2008	751,888 (9098 autism; 742,790 controls)	9–20 years	Maternal history of asthma identified via the Danish National Prescription Registry, or if they received the following asthma medications: the β2AA, inhaled glucocorticoids (R03BA), inhaled anticholinergics (R03BB), theophylline (R03DA), oral leukotriene receptor antagonists (R03DC), systemic steroid (H02AB), adrenergic in combination with corticosteroids, or other drugs (R03AK).	Autism cases were identified from the Danish Psychiatric Central Register23 and the Danish National Patient Register (DNPR), using the ICD‐10 classifications for childhood autism, Atypical autism, Asperger syndrome, other pervasive developmental disorder, and pervasive developmental disorder unspecified.	NA	Sex, paternal age, maternal age, parity, maternal smoking during pregnancy, maternal educational level, cohabitation status, maternal income, calendar period of follow‐up, family history of psychiatric disorders, maternal history of asthma before childbirth, and preterm birth.	Good	Children born to women who used β2AA during pregnancy (especially during the 2nd trimester) had increased odds of autism in later life, which the authors reported was more likely due to underlying maternal diseases rather than the exposure to β2AA itself.
Yu 2023^a^	USA	Cohort	Infants born 2001–2014	318,751 (4559 autism; 314,192 controls)	Birth: 5 years	Asthma was categorized based on the ICD‐9 code 493 and the usage of medication for asthma during pregnancy.	Identified by ICD‐9 codes and ICD‐10 codes on electronic medical records	Non‐Hispanic white: autism = 21%; controls = 25.5%	Child sex, maternal age at delivery, parity, and maternal history of severe comorbidities [> 1 diagnosis of heart, lung, kidney, liver disease, or cancer], birth years, potential air pollution seasonality, maternal race/ethnicity, maternal education and neighborhood disadvantage index at birth.	Good	The hazard ratio after adjusting for covariates indicated a significant association between maternal asthma and child autism.

^a^ Pairs represent studies with overlapping cohorts.

Nine of the 19 included studies found that maternal asthma was not associated with increased odds of child autism (Croen et al. 2011; Gidaya et al. 2016; Langridge et al. 2013; Leonard et al. 2006; Lyall et al. 2014; Micali et al. 2004; Mouridsen et al. 2007; Singer et al. 2017, 2016); however, one of these studies did find increased odds in child autism for asthma medication use during pregnancy compared to women who did not use asthma medication (Gidaya et al. 2016). The remaining 10 studies reported statistically significant increased odds for child autism with maternal asthma (Carter et al. 2023; Casey et al. 2022; Croen et al. 2023, 2005, 2019; Gong et al. 2019; Grivas et al. 2022; Hisle‐Gorman et al. 2018; Su et al. 2017; Yu et al. 2023). Of the eight studies that examined asthma medication use during pregnancy, three studies found increased odds of child autism (Croen et al. 2019; Gidaya et al. 2016; Su et al. 2017), whereas others found no increased odds (Croen et al. 2023, 2011, 2005; Gong et al. 2019; Hisle‐Gorman et al. 2018).

### 3.3 Quality Assessment

Three studies were assessed to be of poor quality, 1 fair quality, and 15 good quality (Table S1). Studies considered poor quality did not control for any confounding variables (Casey et al. 2022; Grivas et al. 2022; Singer et al. 2016). The fair quality study identified cases based on ICD codes with no reference to the primary record, and their comparison group was selected from within the same medical program as the cases (Croen et al. 2005).

### 3.4 Maternal Asthma and Child Autism Meta‐Analyses

#### 3.4.1 Any History of Maternal Asthma

Of the 19 included studies, 14 were eligible for a meta‐analysis examining the association between any history of maternal asthma and child autism. The remaining five studies were not included due to overlapping cohorts. Studies included were selected based on quantitative data being provided or larger sample sizes.

**FIGURE 2:**
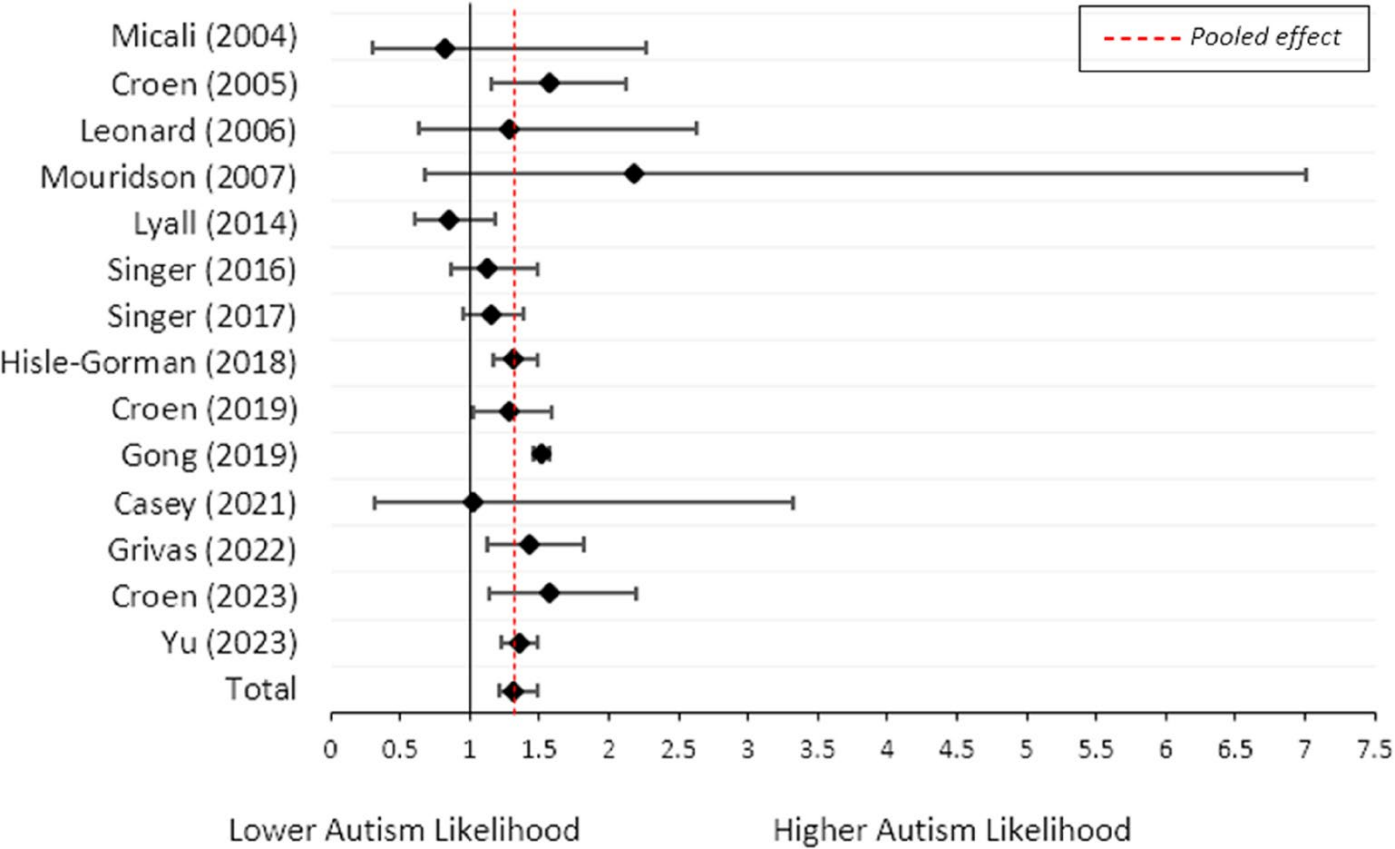
Forest plot showing the odds of child autism when there is any history of maternal asthma. *Note:* The proportions informing this meta‐analysis, the resulting odds ratios, and the total sample size can be found in Table S2.

Any history of maternal asthma was associated with increased odds of child autism (OR = 1.32; 95% CI = 1.21, 1.44; *I* ^2^ = 61%, *n* = 14; Figure 2). Egger's statistical test of the funnel plot (Figure S1) was not significant (*z* = −1.25, *p* = 0.21), suggesting no evidence of publication bias.

A high level of heterogeneity was observed (*I* ^2^ = 61%, *p* = 0.002). To identify the source of heterogeneity and test the robustness of the synthesized results, a sensitivity analysis was conducted by individually removing each study. Gong et al. (2019) contributed a significant amount of heterogeneity to the meta‐analysis, with Lyall et al. (2014) slightly increasing the heterogeneity. Removing both studies showed an almost identical result (OR = 1.32; 95% CI = 1.24, 1.40; *I* ^2^ = 0%, *n* = 12; Table S3). A random effects meta‐analysis of the ORs provided by these studies provided stronger evidence for the association between any history of maternal asthma and child autism (OR = 1.43; 95% CI = 1.38, 1.48; *I* ^2^ = 12%, *n* = 6; Figure S2).

#### 3.4.2 Current Maternal Asthma During Pregnancy

Ten studies were eligible for a meta‐analysis examining the association between current maternal asthma during pregnancy and child autism. Four studies (Carter et al. 2023; Gidaya et al. 2016; Langridge et al. 2013; Su et al. 2017) were excluded due to overlapping cohorts. Five were excluded as they did not identify when the asthma diagnosis was given (Croen et al. 2011, 2005; Grivas et al. 2022; Micali et al. 2004) or did not report any data for current asthma during pregnancy (Mouridsen et al. 2007).

**FIGURE 3:**
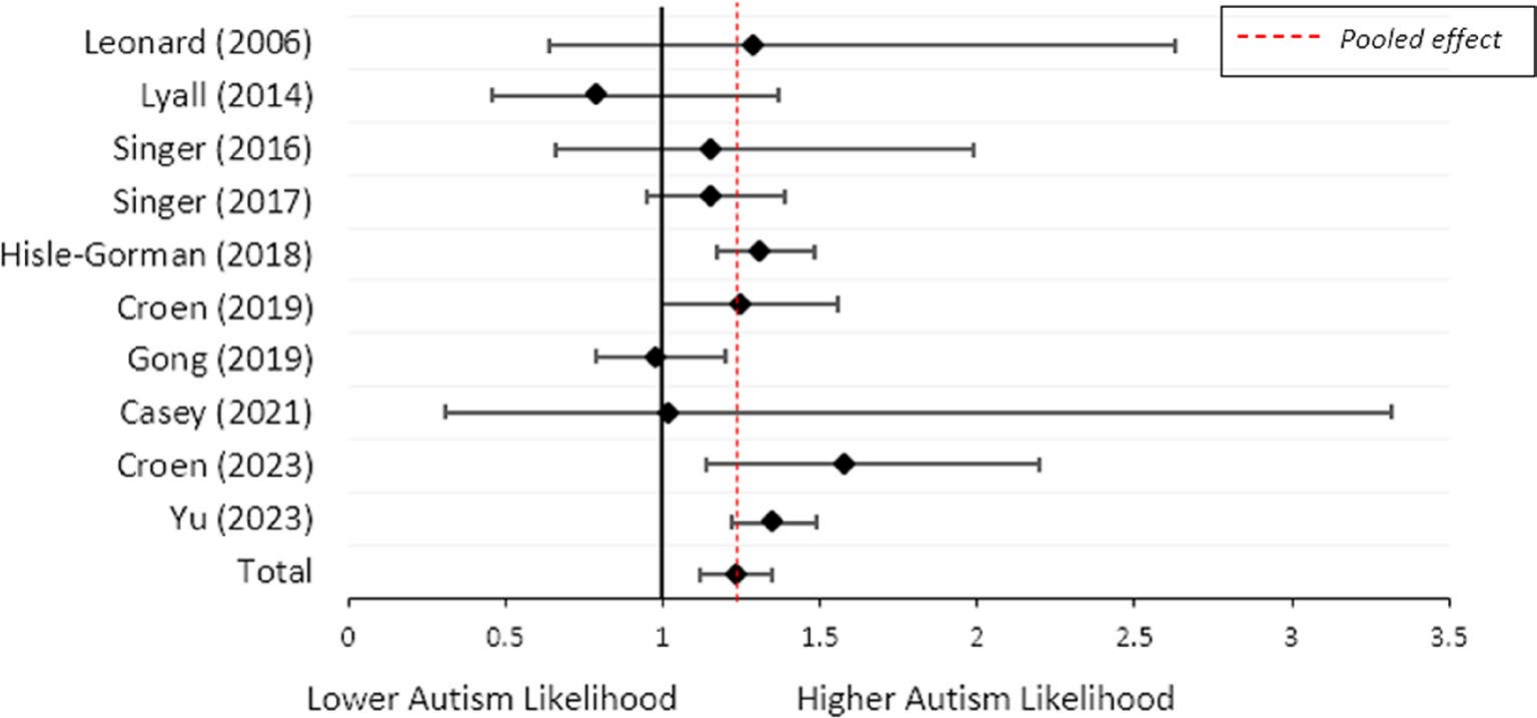
Forest plot showing the odds of child autism when maternal asthma was current during pregnancy, based on the study proportions. *Note:* The proportions informing this meta‐analysis, the resulting odds ratios, and the total sample size can be found in Table S4.

Current asthma during pregnancy was associated with increased odds of child autism (OR = 1.23; 95% CI = 1.12, 1.35; *I* ^2^ = 35%, *n* = 10; Figure 3). Egger's statistical test of the funnel plot (Figure S3) was not significant (*z* = −0.98, *p* = 0.33), suggesting there is no evidence of publication bias in this meta‐analysis.

A moderate level of heterogeneity was observed (*I* ^2^ = 35%, *p* = 0.13). Removing one study (Gong et al. 2023), which contributed to all heterogeneity, showed that the positive association between current asthma during pregnancy and child autism was still evident (OR = 1.30; 95% CI = 1.22, 1.39; *I* ^2^ = 0%, *n* = 9; Table S5). Results suggested a stronger association when ORs were combined (OR = 1.42; 95% CI = 1.25, 1.61; *I* ^2^ = 26%, *n* = 2; Figure S4).

#### 3.4.3 Asthma Medication Use in Pregnancy and Child Autism

Five studies provided data on medication use in pregnancy. The studies were identified for inclusion based on a confirmed asthma diagnosis, and medication explicitly used to treat asthma, comparability of class of medications, and period of use during pregnancy. As the studies used different comparison groups, two meta‐analyses were conducted. One compared the effect of asthma medication use across women with asthma, whereas the other compared women with asthma using medication against those without asthma. An overview of the included studies, including their definition for exposure to asthma, and types of medication used are shown in Table S6.

**FIGURE 4:**
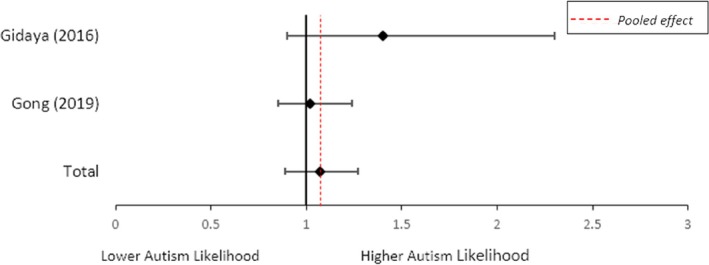
Forest plot showing the odds of child autism when asthma medication was used during pregnancy for women with asthma. *Note:* This meta‐analysis combined the odds ratios of medication use in pregnancy for women with asthma using medication, against women with asthma who did not use medication.

**Women with asthma who used medication vs. women with asthma who did not.** A random effects meta‐analysis identified no significant association between women with asthma who used asthma medication during pregnancy and women with asthma who did not use asthma medication and child autism (OR = 1.07; 95% CI = 0.89, 1.27; *I* ^2^ = 34%, *n* = 2; Figure 4).

**FIGURE 5:**
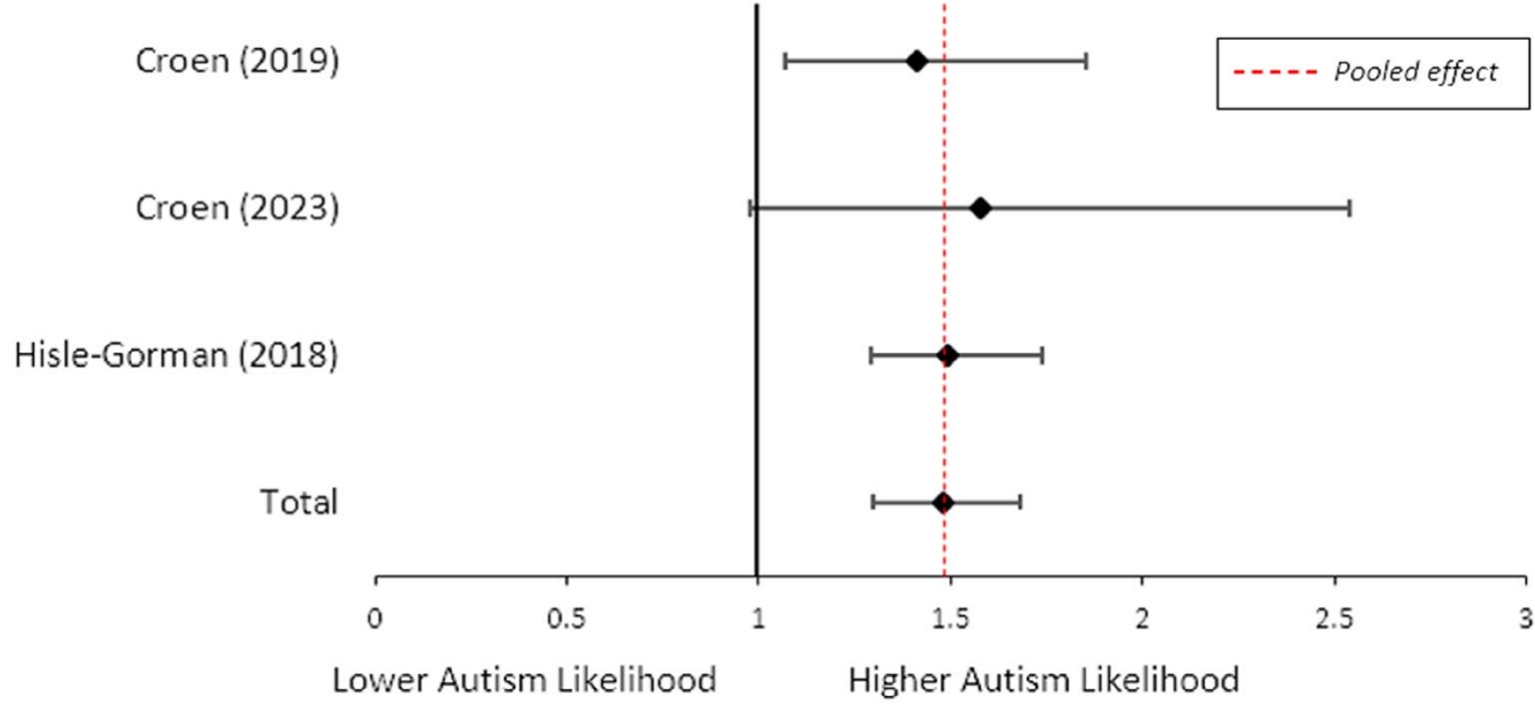
Forest plot showing the odds of child autism when women with asthma used medication during pregnancy compared with women without asthma.

**Women with asthma who used medication vs. women without asthma.** A random effects meta‐analysis identified increased odds of child autism when women with asthma used medication during pregnancy, compared to women without asthma (OR = 1.48; 95% CI = 1.30, 1.68; *I* ^2^ = 0%, *n* = 3; Figure 5).

## 4 Discussion

To our knowledge, this is the first meta‐analysis to evaluate the association between maternal asthma and asthma medication use in pregnancy with child autism. Nineteen studies were eligible for inclusion in the systematic review, with up to 14 studies included in each of the four meta‐analyses. The results consistently suggest that maternal asthma may be associated with increased odds of child autism. While we cannot conclude whether asthma medication directly influences neurodevelopmental outcomes, such differences are more likely due to the severity of asthma or exacerbations during pregnancy, as the likelihood of neurodevelopmental differences was similar among children whose mothers used asthma medication and those who did not, suggesting no significant association with asthma medication use. We have also explored, for the first time, four distinct questions related to the presence of maternal asthma during pregnancy and asthma medication use in pregnancy and child autism.

### 4.1 Maternal Asthma and Child Autism

Here, the evidence showed that having any history of maternal asthma was associated with 32% increased odds of autism, which increased when adjusted ORs were combined (43% odds) and remained when removing studies responsible for most heterogeneity (32% odds). Specifically, when asthma was current during pregnancy, there was a 23% increased odds of child autism, which increased when adjusted ORs were combined (42% odds) and when removing studies responsible for most heterogeneity (30% odds). This data contributes to a growing body of literature that shows associations between maternal asthma and child neurodevelopmental differences. Beyond autism, maternal asthma has been associated with a 50% increase in the odds of child ADHD (OR = 1.5; 95% CI = 1.4–1.6) even when adjusting for parental ADHD (Instanes et al. 2017). Additionally, it has been suggested that asthma exacerbations before, during pregnancy, and after birth further increase the likelihood of child ADHD (Liu et al. 2019). However, this finding was contradicted by Ho et al. (2024), who found acute maternal asthma exacerbations during pregnancy were not associated with a significantly increased likelihood of child ADHD when a sibling comparison method was used. Nevertheless, Ho et al. (2024) found mothers with asthma still had a higher likelihood of child ADHD, autism, and other immunological conditions, with maternal asthma (HR = 1.36, 95% CI = 1.31, 1.40) more strongly associated with child ADHD than paternal asthma (HR = 1.10, 95% CI = 1.05, 1.14). Therefore, it remains unclear whether asthma exacerbations or asthma severity may be associated with child neurodevelopmental outcomes differently. It is likely, however, that mild asthma may remain undiagnosed in some women so effects may be underestimated.

Similarly, child autism has been linked to other maternal inflammatory conditions. Meta‐analyses have found maternal (and not paternal) rheumatoid arthritis (Sun et al. 2022; Zhu et al. 2020), and systemic lupus erythematosus in western populations (Zhu et al. 2020) are associated with a higher likelihood of child autism. Whereas autoimmune or inflammatory disorders are associated with a higher likelihood of child autism and ADHD (Ellul et al. 2022). Hence, this study contributes to evidence suggesting that the maternal immune system may play a role in the neurodevelopmental trajectory of infants during pregnancy.

### 4.2 Maternal Medication Use for Asthma and Child Autism

We could not determine whether there is an association between maternal asthma medication use and child autism, separate from the effects of asthma itself. When women used medication for asthma during pregnancy, there was a 48% increased odds of child autism, compared with women without asthma. However, there was no significant difference in the rate of autism among children born to mothers with asthma who used asthma medication, when compared to women with asthma who did not use asthma medication. This suggests that medication use for asthma during pregnancy does not alter the neurodevelopmental outcomes for these children. However, these findings should be interpreted with caution considering only two to three studies, and a small number of participants in each, informed these meta‐analyses. Results may also be confounded by indication, as women with more severe asthma are more likely to use medication, and both the severity of the women's asthma and the effectiveness of the asthma medication in controlling the asthma symptoms are unknown. Additionally, Robijn et al. (2019) found that despite pregnant women with asthma filling their scripts, they are more likely to either not use their medication, or not use it as prescribed, particularly during the first trimester of pregnancy. Therefore, this meta‐analysis may be underpowered and unlikely to have captured the true effect of asthma medication use on child autism. Nonetheless, our findings are similar to that of Liu et al. (2019), where maternal asthma is associated with child ADHD, while asthma medication use during pregnancy is not.

### 4.3 Strengths and Limitations

The inclusion of 19 studies in the systematic review, and up to 14 studies in the meta‐analyses, with no substantial publication bias and robustness across each of the sensitivity analyses, are strengths of this study. The outcomes also remained unchanged after removing all heterogeneity between the studies, and with all poorly assessed studies removed.

General limitations were identified across the studies. All included studies have been conducted in WEIRD (western, educated, industrialized, rich, democratic) countries, with predominantly white participants. As autism rates vary across sociodemographic and racial groups (Zeidan et al. 2022), the lack of representation across populations limits the generalizability of these findings. Furthermore, several studies that found no association between maternal asthma and child autism were underpowered. Many reported on a small number of autistic (Casey et al. 2022), or exposed participants (e.g., Croen et al. 2019; Leonard et al. 2006; Micali et al. 2004; Mouridsen et al. 2007). Another indicated a low response rate in their comparison group resulting in measurement bias (Micali et al. 2004). A reliance on self‐report (Casey et al. 2022; Singer et al. 2017) and no case confirmation (Langridge et al. 2013) may have also contributed to the misclassification of participants and the lack of significant findings.

Another key limitation that was also highlighted by Seker et al. (2023) is the lack of studies controlling for the heritability of autism. Autism is highly heritable, with meta‐analyses suggesting heritability rates between 64% and 91% (Tick et al. 2016). A family history of other neurodevelopmental conditions is also associated with higher odds of child autism (George et al. 2014). Here, none of the included studies controlled for parental neurodivergence. Consequently, our results should be interpreted with some caution as the effect of maternal asthma on child autism may be overestimated here, as other genetic influences may explain some of this interaction. It is imperative that future studies control for genetic confounders, to ensure an accurate understanding of the association between maternal asthma and child autism.

We were also unable to investigate both trimester effects and asthma severity on child outcomes, due to insufficient data. Studies consistently reported a lack of information in medical records about whether asthma was active or dormant during pregnancy (Croen et al. 2019; Yu et al. 2023), and some did not specify which medication was used, or their duration of use (Croen et al. 2011, 2019; Hisle‐Gorman et al. 2018; Su et al. 2017). Furthermore, a reliance on prescription dispensing records meant that we could not determine the actual dose, frequency, and duration of medications taken. Additionally, as Gong et al. (2019) did not retrieve dispensing records for all participants, their effect of medication use in pregnancy may be underestimated. Overall, even when asthma medication use is known, there are no records on whether the medication was effective in managing asthma symptoms. Not having this level of data prevented us from accurately distinguishing between disease and treatment effects, and from analyzing the trimester effects of each medication used.

### 4.4 Future Directions

This study suggests that women with asthma may be more likely to have an autistic child. However, some caution is warranted in how these findings are interpreted. Considering the high heritability rate of autism, if a child is identified as autistic, it is possible that the mother, or other immediate family members, may also be autistic or have autistic characteristics. This is also plausible considering the gender differences in the core characteristics associated with autism, which tend to result in autism diagnoses being missed or delayed in females (Bargiela et al. 2016; Fusar‐Poli et al. 2022). Furthermore, the high co‐occurrence rate between autism and allergic conditions such as asthma should not be dismissed (Chua et al. 2020), as the mother's asthma may independently be associated with autism characteristics, which could be a contributor toward the increased likelihood of autism in the child. Therefore, we do not confer causation; rather, we highlight that there are complexities in the association between maternal asthma and autism. It is important that future work controls for autism heritability and addresses the limitations surrounding medication use discussed. More longitudinal studies that rely on current, objective diagnostic measures for both asthma and autism, and which distinguish between the treatment effects, shared genetic links, and account for other confounders, are needed. Future studies that differentiate between asthma medications used and their independent effects on neurodevelopment would also be valuable. There is the possibility of confounding by indication, which we were not able to investigate further, as there was no data available to deduce asthma severity or what dose, frequency, or duration of medication was used for these participants. These further investigations will provide a better understanding of the relationship between maternal asthma and child autism.

## 5 Conclusion

There is converging evidence that maternal asthma can be associated with increased odds of neurodevelopmental differences in the developing child, such as autism, for which inflammatory processes have been suggested as a potential mechanism (e.g., Han et al. 2021; Seker et al. 2023). This systematic review and meta‐analyses have found that any history of maternal asthma and current asthma during pregnancy is associated with child autism. However, different methodologies among studies mean we cannot infer a causal relationship between maternal asthma and child autism, as more robust longitudinal studies are needed to distinguish between potential confounders. There is also a paucity of research examining the trimester effects of asthma, asthma exacerbations, asthma severity, and asthma medication use in pregnancy, which could be addressed in future prospective studies. Importantly, future research should control for genetic and familial confounders, and whether multiple immune activation triggers further increase this likelihood, as this may contribute to a better understanding of the underlying mechanisms that influence child neurodevelopment.

## Author Contributions

The authors' responsibilities were as follows: V.E.M., O.W., and A.L. conceived of the study and developed the study methodology. S.H., R.v.d.S., P.D., and O.W. formally reviewed the data. R.v.d.S. analyzed the data. V.E.M. acquired the study resources. S.H., R.v.d.S., and O.W. curated the data. R.v.d.S. and V.E.M. wrote the manuscript draft. R.v.d.S., V.E.M., and O.W. reviewed and edited the manuscript. V.E.M. and O.W. supervised the research. All authors read and approved the final manuscript.

## Disclosure

The authors have nothing to report.

## Conflicts of Interest

The authors declare no conflicts of interest.

## Supporting information


**Data S1.** Supporting Information.

## Data Availability

Published data used for the meta‐analyses are available upon request.

## References

[aur70071-bib-0001] American Psychiatric Association . 2000. Diagnostic and Statistical Manual of Mental Disorders. 4th ed. American Psychiatric Association.

[aur70071-bib-0002] American Psychiatric Association . 2013. Diagnostic and Statistical Manual of Mental Disorders. 5th ed. American Psychiatric Association.

[aur70071-bib-0003] Bargiela, S. , R. Steward , and W. Mandy . 2016. “The Experiences of Late‐Diagnosed Women With Autism Spectrum Conditions: An Investigation of the Female Autism Phenotype.” Journal of Autism and Developmental Disorders 46, no. 10: 3281–3294. 27457364 10.1007/s10803-016-2872-8 PMC5040731

[aur70071-bib-0004] Carter, S. A. , J. C. Lin , T. Chow , et al. 2023. “Maternal Obesity, Diabetes, Preeclampsia, and Asthma During Pregnancy and Likelihood of Autism Spectrum Disorder With Gastrointestinal Disturbances in Offspring.” Autism 27: 916–926. 10.1177/13623613221118430. 36062479 PMC9984567

[aur70071-bib-0005] Casey, S. , M. Carter , A. M. Looney , et al. 2022. “Maternal Mid‐Gestation Cytokine Dysregulation in Mothers of Children With Autism Spectrum Disorder.” Journal of Autism and Developmental Disorders 52, no. 9: 3919–3932. 10.1007/s10803-021-05271-7. 34505185 PMC9349096

[aur70071-bib-0006] Chua, R. X. Y. , M. J. Y. Tay , D. S. Q. Ooi , et al. 2020. “Understanding the Link Between Allergy and Neurodevelopmental Disorders: A Current Review of Factors and Mechanisms.” Frontiers in Neurology 11: 603571. 10.3389/fneur.2020.603571. 33658968 PMC7917177

[aur70071-bib-0007] Croen, L. A. , J. L. Ames , Y. Qian , et al. 2023. “Inflammatory Conditions During Pregnancy and Risk of Autism and Other Neurodevelopmental Disorders.” Biological Psychiatry Global Open Science 4, no. 1: 39–50. 10.1016/j.bpsgos.2023.09.008. 38045769 PMC10689278

[aur70071-bib-0008] Croen, L. A. , S. L. Connors , M. Matevia , Y. Qian , C. Newschaffer , and A. W. Zimmerman . 2011. “Prenatal Exposure to β2‐Adrenergic Receptor Agonists and Risk of Autism Spectrum Disorders.” Journal of Neurodevelopmental Disorders 3, no. 4: 307–315. 10.1007/s11689-011-9093-4. 21874331 PMC3261266

[aur70071-bib-0009] Croen, L. A. , J. K. Grether , C. K. Yoshida , R. Odouli , and J. Van de Water . 2005. “Maternal Autoimmune Diseases, Asthma and Allergies, and Childhood Autism Spectrum Disorders: A Case‐Control Study.” Archives of Pediatrics & Adolescent Medicine 159, no. 2: 151–157. 10.1001/archpedi.159.2.151. 15699309

[aur70071-bib-0010] Croen, L. A. , Y. Qian , P. Ashwood , et al. 2019. “Family History of Immune Conditions and Autism Spectrum and Developmental Disorders: Findings From the Study to Explore Early Development.” Autism Research 12, no. 1: 123–135. 10.1002/aur.1979. 30095240 PMC6467644

[aur70071-bib-0011] Davies, G. , S. Jordan , D. Thayer , D. Tucker , and I. Humphreys . 2020. “Medicines Prescribed for Asthma, Discontinuation and Perinatal Outcomes, Including Breastfeeding: A Population Cohort Analysis.” PLoS One 15, no. 12: e0242489. 10.1371/journal.pone.0242489. 33296383 PMC7725302

[aur70071-bib-0012] Egger, M. , G. Davey Smith , M. Schneider , and C. Minder . 1997. “Bias in Meta‐Analysis Detected by a Simple, Graphical Test.” BMJ (Clinical Research Ed.) 315, no. 7109: 629–634. 10.1136/bmj.315.7109.629. PMC2127453 9310563

[aur70071-bib-0013] Ellul, P. , E. Acquaviva , H. Peyre , et al. 2022. “Parental Autoimmune and Autoinflammatory Disorders as Multiple Risk Factors for Common Neurodevelopmental Disorders in Offspring: A Systematic Review and Meta‐Analysis.” Translational Psychiatry 12, no. 1: 112. 10.1038/s41398-022-01843-y. 35304436 PMC8933391

[aur70071-bib-0014] Flannery, K. A. , and J. Liederman . 1994. “A Test of the Immunoreactive Theory for the Origin of Neurodevelopmental Disorders in the Offspring of Women With Immune Disorder.” Cortex 30, no. 4: 635–646. 10.1016/S0010-9452(13)80240-7. 7535215

[aur70071-bib-0015] Fusar‐Poli, L. , N. Brondino , P. Politi , and E. Aguglia . 2022. “Missed Diagnoses and Misdiagnoses of Adults With Autism Spectrum Disorder.” European Archives of Psychiatry and Clinical Neuroscience 272, no. 2: 187–198. 10.1007/s00406-020-01189-w. 32892291 PMC8866369

[aur70071-bib-0016] George, B. , M. S. R. Padmam , M. K. C. Nair , M. L. Leena , and P. S. S. Russell . 2014. “CDC Kerala 13: Antenatal, Natal and Postnatal Factors Among Children (2–6 y) With Autism—A Case Control Study.” Indian Journal of Pediatrics 81, no. 2: 133–137. 10.1007/s12098-014-1594-1. 25338492

[aur70071-bib-0017] Gidaya, N. B. , B. K. Lee , I. Burstyn , Y. Michael , C. J. Newschaffer , and E. L. Mortensen . 2016. “In Utero Exposure to β‐2‐Adrenergic Receptor Agonist Drugs and Risk for Autism Spectrum Disorders.” Pediatrics 137, no. 2: e20151316. 10.1542/peds.2015-1316. 26738885

[aur70071-bib-0018] Gong, T. , C. Lundholm , S. Lundstrom , R. Kuja‐Halkola , M. J. Taylor , and C. Almqvist . 2023. “Understanding the Relationship Between Asthma and Autism Spectrum Disorder: A Population‐Based Family and Twin Study.” Psychological Medicine 53, no. 7: 3096–3104. 10.1017/S0033291721005158. 35388771 PMC10235668

[aur70071-bib-0019] Gong, T. , C. Lundholm , G. Rejnö , et al. 2019. “Parental Asthma and Risk of Autism Spectrum Disorder in Offspring: A Population and Family‐Based Case‐Control Study.” Clinical & Experimental Allergy 49, no. 6: 883–891. 10.1111/cea.13353. 30742718 PMC6849600

[aur70071-bib-0020] Gordon, M. , K. R. Niswander , H. Berendes , and A. G. Kantor . 1970. “Fetal Morbidity Following Potentially Anoxigenic Obstetric Conditions: VII. Bronchial Asthma.” American Journal of Obstetrics and Gynecology 106, no. 3: 421–429. 10.1016/0002-9378(70)90371-6. 5410878

[aur70071-bib-0021] Grivas, G. , R. E. Frye , and J. Hahn . 2022. “Maternal Risk Factors Vary Between Subpopulations of Children With Autism Spectrum Disorder.” Autism Research 15, no. 11: 2038–2055. 10.1002/aur.2809. 36065595 PMC9637779

[aur70071-bib-0022] Han, V. X. , S. Patel , H. F. Jones , et al. 2021. “Maternal Acute and Chronic Inflammation in Pregnancy Is Associated With Common Neurodevelopmental Disorders: A Systematic Review.” Translational Psychiatry 11, no. 1: 71. 10.1038/s41398-021-01198-w. 33479207 PMC7820474

[aur70071-bib-0023] Hansen, C. , P. Joski , H. Freiman , et al. 2013. “Medication Exposure in Pregnancy Risk Evaluation Program: The Prevalence of Asthma Medication Use During Pregnancy.” Maternal and Child Health Journal 17, no. 9: 1611–1621. 10.1007/s10995-012-1173-x. 23108737 PMC3797257

[aur70071-bib-0024] Higgins, J. P. T. , S. G. Thompson , J. J. Deeks , and D. G. Altman . 2003. “Measuring Inconsistency in Meta‐Analyses.” BMJ (Clinical Research Ed.) 327, no. 7414: 557. 10.1136/bmj.327.7414.557. PMC192859 12958120

[aur70071-bib-0025] Hisle‐Gorman, E. , A. Susi , T. Stokes , G. Gorman , C. Erdie‐Lalena , and C. M. Nylund . 2018. “Prenatal, Perinatal, and Neonatal Risk Factors of Autism Spectrum Disorder.” Pediatric Research 84, no. 2: 190–198. 10.1038/pr.2018.23. 29538366

[aur70071-bib-0026] Ho, Y.‐F. , Y.‐L. Chen , R. Stewart , T.‐C. Hsu , and V. C.‐H. Chen . 2024. “Maternal Asthma and Asthma Exacerbation During Pregnancy and Attention‐Deficit/Hyperactivity Disorder in Offspring: A Population‐Based Cohort Study.” European Child & Adolescent Psychiatry 33, no. 11: 3841–3848. 10.1007/s00787-024-02426-6. 38600406

[aur70071-bib-0027] Innovation, V. H. 2023. “Covidence Systematic Review Software.” www.covidence.org.

[aur70071-bib-0028] Instanes, J. T. , A. Halmøy , A. Engeland , J. Haavik , K. Furu , and K. Klungsøyr . 2017. “Attention‐Deficit/Hyperactivity Disorder in Offspring of Mothers With Inflammatory and Immune System Diseases.” Biological Psychiatry 81, no. 5: 452–459. 10.1016/j.biopsych.2015.11.024. 26809250

[aur70071-bib-0029] Langridge, A. T. , E. J. Glasson , N. Nassar , et al. 2013. “Maternal Conditions and Perinatal Characteristics Associated With Autism Spectrum Disorder and Intellectual Disability.” PLoS One 8, no. 1: e50963. 10.1371/journal.pone.0050963. 23308096 PMC3538698

[aur70071-bib-0030] Leonard, H. , N. de Klerk , J. Bourke , and C. Bower . 2006. “Maternal Health in Pregnancy and Intellectual Disability in the Offspring: A Population‐Based Study.” Annals of Epidemiology 16, no. 6: 448–454. 10.1016/j.annepidem.2005.05.002. 16182562

[aur70071-bib-0031] Liu, X. , S. Dalsgaard , T. Munk‐Olsen , J. Li , R. J. Wright , and N. C. Momen . 2019. “Parental Asthma Occurrence, Exacerbations and Risk of Attention‐Deficit/Hyperactivity Disorder.” Brain, Behavior, and Immunity 82: 302–308. 10.1016/j.bbi.2019.08.198. 31476415 PMC7408292

[aur70071-bib-0032] Lyall, K. , P. Ashwood , J. Van de Water , and I. Hertz‐Picciotto . 2014. “Maternal Immune‐Mediated Conditions, Autism Spectrum Disorders, and Developmental Delay.” Journal of Autism and Developmental Disorders 44, no. 7: 1546–1555. 10.1007/s10803-013-2017-2. 24337796 PMC4104679

[aur70071-bib-0033] Magalhães, E. S. , F. Pinto‐Mariz , S. Bastos‐Pinto , A. T. Pontes , E. A. Prado , and L. C. deAzevedo . 2009. “Immune Allergic Response in Asperger Syndrome.” Journal of Neuroimmunology 216, no. 1: 108–112. 10.1016/j.jneuroim.2009.09.015. 19840888

[aur70071-bib-0034] Mann, J. R. , S. McDermott , H. Bao , J. Hardin , and A. Gregg . 2010. “Pre‐Eclampsia, Birth Weight, and Autism Spectrum Disorders.” Journal of Autism and Developmental Disorders 40, no. 5: 548–554. 10.1007/s10803-009-0903-4. 19936906

[aur70071-bib-0035] Micali, N. , S. Chakrabarti , and E. Fombonne . 2004. “The Broad Autism Phenotype: Findings From an Epidemiological Survey.” Autism 8, no. 1: 21–37. 10.1177/1362361304040636. 15070545

[aur70071-bib-0036] Mouridsen, S. E. , B. Rich , T. Isager , and N. J. Nedergaard . 2007. “Autoimmune Diseases in Parents of Children With Infantile Autism: A Case—Control Study.” Developmental Medicine and Child Neurology 49, no. 6: 429–432. 10.1111/j.1469-8749.2007.00429.x. 17518928

[aur70071-bib-0037] Murphy, V. , P. Gibson , R. Smith , and V. Clifton . 2005. “Asthma During Pregnancy: Mechanisms and Treatment Implications.” European Respiratory Journal 25: 731–750. 10.1183/09031936.05.00085704. 15802351

[aur70071-bib-0038] Page, M. J. , J. P. Higgins , and J. A. Sterne . 2023. “Chapter 13: Assessing Risk of Bias due to Missing Results in a Synthesis.” In Cochrane Handbook for Systematic Reviews of Interventions Version 6.4, edited by J. P. T. Higgins , J. Thomas , J. Chandler , et al. Cochrane. Updated August 2023. https://training.cochrane.org/handbook/.

[aur70071-bib-0039] Page, M. J. , J. E. McKenzie , P. M. Bossuyt , et al. 2021. “The PRISMA 2020 Statement: An Updated Guideline for Reporting Systematic Reviews.” Journal of Clinical Epidemiology 134: 178–189. 10.1016/j.jclinepi.2021.03.001. 33789819

[aur70071-bib-0040] Patel, S. , R. C. Dale , D. Rose , et al. 2020. “Maternal Immune Conditions Are Increased in Males With Autism Spectrum Disorders and Are Associated With Behavioural and Emotional but Not Cognitive Co‐Morbidity.” Translational Psychiatry 10, no. 1: 286. 10.1038/s41398-020-00976-2. 32796821 PMC7429839

[aur70071-bib-0041] Patel, S. , A. Masi , R. C. Dale , et al. 2017. “Social Impairments in Autism Spectrum Disorder Are Related to Maternal Immune History Profile.” Molecular Psychiatry 23, no. 8: 1794–1797. 10.1038/mp.2017.201. 28993711

[aur70071-bib-0042] Review Manager (RevMan) . 2020. “The Cochrane Collaboration (Version 5.4.1).”

[aur70071-bib-0043] Robijn, A. L. , S. M. Harvey , M. E. Jensen , et al. 2024. “Adverse Neonatal Outcomes in Pregnant Women With Asthma: An Updated Systematic Review and Meta‐Analysis.” International Journal of Gynecology & Obstetrics 166: 596–606. 10.1002/ijgo.15407. 38327138

[aur70071-bib-0044] Robijn, A. L. , M. E. Jensen , K. McLaughlin , P. G. Gibson , and V. E. Murphy . 2019. “Inhaled Corticosteroid Use During Pregnancy Among Women With Asthma: A Systematic Review and Meta‐Analysis.” Clinical and Experimental Allergy 49, no. 11: 1403–1417. 10.1111/cea.13474. 31357230

[aur70071-bib-0045] Rocklin, R. E. 2011. “Asthma, Asthma Medications and Their Effects on Maternal/Fetal Outcomes During Pregnancy.” Reproductive Toxicology 32, no. 2: 189–197. 10.1016/j.reprotox.2011.05.023. 21684328

[aur70071-bib-0046] Sawicki, E. , K. Stewart , S. Wong , L. Leung , E. Paul , and J. George . 2011. “Medication Use for Chronic Health Conditions by Pregnant Women Attending an Australian Maternity Hospital.” Australian and New Zealand Journal of Obstetrics and Gynaecology 51, no. 4: 333–338. 10.1111/j.1479-828X.2011.01312.x. 21806573

[aur70071-bib-0047] Schatz, M. , M. P. Dombrowski , R. Wise , et al. 2004. “The Relationship of Asthma Medication Use to Perinatal Outcomes.” Journal of Allergy and Clinical Immunology 113, no. 6: 1040–1045. 10.1016/j.jaci.2004.03.017. 15208581

[aur70071-bib-0048] Seker, A. , A. Qirko‐Gurakuqi , M. Tabaku , K. R. P. Javate , and I. Rathwell . 2023. “Maternal Atopic Conditions and Autism Spectrum Disorder: A Systematic Review.” European Child & Adolescent Psychiatry 33: 3727–3737. 10.1007/s00787-023-02285-7. 37661216 PMC11588786

[aur70071-bib-0049] Singer, A. B. , I. Burstyn , M. Thygesen , P. B. Mortensen , M. D. Fallin , and D. E. Schendel . 2017. “Parental Exposures to Occupational Asthmagens and Risk of Autism Spectrum Disorder in a Danish Population‐Based Case‐Control Study.” Environmental Health 16, no. 1: 31. 10.1186/s12940-017-0230-8. 28359263 PMC5374665

[aur70071-bib-0050] Singer, A. B. , G. C. Windham , L. A. Croen , et al. 2016. “Maternal Exposure to Occupational Asthmagens During Pregnancy and Autism Spectrum Disorder in the Study to Explore Early Development.” Journal of Autism and Developmental Disorders 46, no. 11: 3458–3468. 10.1007/s10803-016-2882-6. 27511194 PMC5073112

[aur70071-bib-0051] StataCorp . 2023. Stata Statistical Software: Release 18. StataCorp LLC.

[aur70071-bib-0052] Su, X. , W. Yuan , J. Chen , et al. 2017. “Prenatal Exposure to β2‐Adrenoreceptor Agonists and the Risk of Autism Spectrum Disorders in Offspring.” Pharmacoepidemiology and Drug Safety 26, no. 7: 812–818. 10.1002/pds.4214. 28422339

[aur70071-bib-0053] Sun, C. K. , Y. S. Cheng , I. W. Chen , et al. 2022. “Impact of Parental Rheumatoid Arthritis on Risk of Autism Spectrum Disorders in Offspring: A Systematic Review and Meta‐Analysis.” Frontiers in Medicine 9: 1052806. 10.3389/fmed.2022.1052806. 36438039 PMC9687371

[aur70071-bib-0054] Tick, B. , P. Bolton , F. Happé , M. Rutter , and F. Rijsdijk . 2016. “Heritability of Autism Spectrum Disorders: A Meta‐Analysis of Twin Studies.” Journal of Child Psychology and Psychiatry 57, no. 5: 585–595. 10.1111/jcpp.12499. 26709141 PMC4996332

[aur70071-bib-0055] Wells, G. A. , G. Wells , B. Shea , et al. 2014. “The Newcastle‐Ottawa Scale (NOS) for Assessing the Quality of Nonrandomised Studies in Meta‐Analyses.”

[aur70071-bib-0056] Whalen, O. M. , F. Karayanidis , V. E. Murphy , A. E. Lane , C. A. Mallise , and L. E. Campbell . 2018. “The Effects of Maternal Asthma During Pregnancy on Child Cognitive and Behavioral Development: A Systematic Review.” Journal of Asthma 56, no. 2: 130–141. 10.1080/02770903.2018.1437174. 29482387

[aur70071-bib-0057] World Health Organization . 2011. International Statistical Classification of Diseases and Related Health Problems. World Health Organization.

[aur70071-bib-0058] World Health Organization . 2016. International Statistical Classification of Diseases and Related Health Problems. 10th ed. World Health Organization.

[aur70071-bib-0059] Yu, X. , M. Mostafijur Rahman , S. A. Carter , et al. 2023. “Prenatal Air Pollution, Maternal Immune Activation, and Autism Spectrum Disorder.” Environment International 179: 108148. 10.1016/j.envint.2023.108148. 37595536 PMC10792527

[aur70071-bib-0060] Zeidan, J. , E. Fombonne , J. Scorah , et al. 2022. “Global Prevalence of Autism: A Systematic Review Update.” Autism Research 15, no. 5: 778–790. 10.1002/aur.2696. 35238171 PMC9310578

[aur70071-bib-0061] Zhu, Z. , S. Tang , X. Deng , and Y. Wang . 2020. “Maternal Systemic Lupus Erythematosus, Rheumatoid Arthritis, and Risk for Autism Spectrum Disorders in Offspring: A Meta‐Analysis.” Journal of Autism and Developmental Disorders 50, no. 8: 2852–2859. 10.1007/s10803-020-04400-y. 32034648

