# Supplementary material for: The Association Between Maternal Asthma and Child Autism: A Systematic Review and Meta‐Analysis
Source: Autism Res. 2025 Jun 20;18(8):1630–50. doi: 10.1002/aur.70071 (PMC12384736; doi:10.1002/aur.70071)
Supplement: Supplementary file 1 — Data S1. Supporting Information. [file AUR-18-1630-s001.docx]

**The association between maternal asthma and child autism: A systematic review and meta-analysis**

Contents

Table S1 2

Newcastle-Ottowa Scale assessment of the quality of the included studies. 2

Figure S1 3

Funnel plot of the studies assessing the odds of child autism when there is any history of maternal asthma based on the study proportions. 3

Table S2 3

Proportions, and resulting odds ratios from the any history of maternal asthma and child autism meta-analysis. 3

Table S3 4

Sensitivity analysis of the meta-analysis examining the relationship between any history of maternal asthma and child autism. 4

Figure S2 4

Forest plot showing the odds of child autism when there is any history of maternal asthma based on the study odds ratios. 4

Figure S3 5

Funnel plot of the studies assessing the odds of child autism when maternal asthma was current during pregnancy, based on the study proportions. 5

Table S4 5

Proportions, and resulting odds ratios from the current asthma in pregnancy and child autism meta-analysis. 5

Table S5 6

Sensitivity analysis of the meta-analysis examining the relationship between current maternal asthma in pregnancy and child autism. 6

Figure S4 6

Forest plot showing the odds of child autism when there is current maternal asthma in pregnancy, based on the study odds ratios. 6

Table S6 7

Overview of the studies included in the asthma medication use in pregnancy meta-analyses, including their definition of asthma diagnosis, exposure, and type of medication used during pregnancy. 7

##

## Table S1

### Newcastle-Ottowa Scale assessment of the quality of the included studies.

| **Study** | **Study Type** | **Selection** | **Comparability** | **Exposure/Outcome** | **Total Stars** | **AHQR score** |
| --- | --- | --- | --- | --- | --- | --- |
| Carter 2023 | cohort | 4 | 2 | 3 | 9 | Good |
| Casey 2021 | cohort | 2 | 0 | 1 | 3 | Poor |
| Croen 2005 | case-control | 2 | 2 | 3 | 7 | Fair |
| Croen 2011 | case-control | 4 | 2 | 3 | 9 | Good |
| Croen 2019 | case-control | 4 | 1 | 3 | 8 | Good |
| Croen 2023 | case-control | 3 | 2 | 3 | 8 | Good |
| Gidaya 2016 | case-control | 3 | 2 | 3 | 8 | Good |
| Gong 2019 | case-control | 3 | 2 | 3 | 8 | Good |
| Grivas 2022 | cohort | 4 | 0 | 3 | 7 | Poor |
| Hisle-Gorman 2018 | case-cohort | 3 | 1 | 3 | 7 | Good |
| Langridge 2013 | cohort | 4 | 1 | 3 | 8 | Good |
| Leonard 2006 | cohort | 4 | 1 | 3 | 8 | Good |
| Lyall 2014 | case-control | 4 | 2 | 2 | 8 | Good |
| Micali 2004 | Case-control | 3 | 2 | 2 | 7 | Good |
| Mouridsen 2007 | case-control | 3 | 2 | 3 | 8 | Good |
| Singer 2016 | case-control | 4 | 0 | 2 | 6 | Poor |
| Singer 2017 | case-control | 3 | 2 | 3 | 8 | Good |
| Su 2017 | cohort | 4 | 1 | 3 | 8 | Good |
| Yu 2023 | cohort | 4 | 2 | 3 | 9 | Good |
| **Study** | **Study Type** | **Selection** | **Comparability** | **Outcome** | **Total Stars** | **AHQR score** |

*Note:* This table shows the number of stars awarded for each section of the study when applying the Newcastle-Ottowa Scale. The thresholds for converting the Newcastle-Ottawa scales to the Agency for Health Research and Quality (AHQR) Score (good, fair, and poor) is: Good quality:- 3 or 4 stars in selection domain AND 1 or 2 stars in comparability domain AND 2 or 3 stars in outcome/exposure domain; Fair quality:- 2 stars in selection domain AND 1 or 2 stars in comparability domain AND 2 or 3 stars in outcome/exposure domain; Poor quality:- 0 or 1 star in selection domain OR 0 stars in comparability domain OR 0 or 1 stars in outcome/exposure domain.

## Figure S1

### Funnel plot of the studies assessing the odds of child autism when there is any history of maternal asthma based on the study proportions.

## Table S2

### Proportions, and resulting odds ratios from the any history of maternal asthma and child autism meta-analysis.

## Table S3

### Sensitivity analysis of the meta-analysis examining the relationship between any history of maternal asthma and child autism.

*Note:* This table shows the Odds Ratio (OR), Confidence Internal (CI), and I^2^ statistic for a meta-analysis if that study was removed. Studies marked with a * represent a reduction in heterogeneity if that study is removed from the meta-analysis, i.e., removing Gong (2019) will result in heterogeneity lowering from high to mild, removing Lyall (2014) will lower heterogeneity from high to moderate, and removing both studies results in a meta-analysis with no heterogeneity. The analysis marked with ^ shows the outcome when all studies assessed as poor quality are removed from the analysis.

## Figure S2

### Forest plot showing the odds of child autism when there is any history of maternal asthma based on the study odds ratios.

## Figure S3

### Funnel plot of the studies assessing the odds of child autism when maternal asthma was current during pregnancy, based on the study proportions.

## Table S4

### Proportions, and resulting odds ratios from the current asthma in pregnancy and child autism meta-analysis.

## Table S5

### Sensitivity analysis of the meta-analysis examining the relationship between current maternal asthma in pregnancy and child autism.

*Note:* This table shows the Odds Ratio (OR), Confidence Internal (CI), and I^2^ statistic for a meta-analysis if that study was removed. Studies marked with a * represent a reduction in heterogeneity if that study is removed from the meta-analysis, i.e., removing Gong (2019) removes all heterogeneity in the meta-analysis. ^ Represents the meta-analysis after removing all studies considered to be of poor quality according to the AHQR system of points awarded during the quality assessment shown in Table S1.

## Figure S4

### Forest plot showing the odds of child autism when there is current maternal asthma in pregnancy, based on the study odds ratios.

## Table S6

### Overview of the studies included in the asthma medication use in pregnancy meta-analyses, including their definition of asthma diagnosis, exposure, and type of medication used during pregnancy.
